# Gaps between Adolescent Risk Behaviors and Disclosure during Outpatient Visits

**DOI:** 10.1155/2013/718568

**Published:** 2013-04-24

**Authors:** Linda L. Hill, Melbourne Hovell, Elaine Blumberg, Norma Kelley, Sara Baird, Carol Sipan, Katharine Schmitz, Lawrence Friedman

**Affiliations:** ^1^School of Medicine, University of California, San Diego, CA 92093, USA; ^2^Department of Family and Preventive Medicine, UCSD, 9500 Gilman Drive, MS 0811, La Jolla, CA 92093-0811, USA; ^3^Center for Behavioral Epidemiology and Community Health (C-BEACH), Graduate School of Public Health, San Diego State University, San Diego, CA 92182, USA; ^4^Department of Family Medicine, University of Washington, Seattle, WA 98195-6390, USA

## Abstract

*Objective*. The purpose of this study was to determine the gaps between disclosed high-risk behaviors in low-income, mainly Hispanic youth and the identification of these risks by health care providers. *Methods*. This cross-sectional study included youth 13–19 years old who participated in a study on latent tuberculosis treatment. Youth were interviewed at baseline by bilingual research assistants; the provider visit was assessed by the chart review. *Results*. Of 221 youth, the majority (96%) were identified as Hispanic, 45% were foreign-born, and 46% were male. A total of 399 risk behaviors were revealed to research staff by the participants; only 24 risk behaviors were revealed to providers. *Conclusions*. The majority of risk behaviors based on the chart review were neither queried nor disclosed to the physicians. Physicians providing care to adolescents should consider strategies to improve disclosure as a necessary precursor to interventions.

## 1. Introduction

Adolescence is characterized by evolving personality, impulsivity, rebellion, and deviation from established adult norms, often leading adolescents to take higher personal risks without realizing short- or long-term consequences [1–3]. This behavior is age appropriate to some extent, reflecting the neurodevelopmental chronology of the human brain [4]. This risk taking translates into many arenas of preventable adverse health outcomes including motor vehicle accidents, tobacco use, alcohol and drug use, unsafe sexual activity, and interpersonal violence. The developmental changes that define the teenage years occur across all demographic and culture groups in the USA and globally. According to the USA Census Bureau (2005), 33.9% of the Latino population was under 21 years of age, making health care of Latino youth an important and relevant issue [5]. Yet, Latino youth, in particular, face systemic language and cultural barriers to accessing care and information, making it increasingly difficult to make healthy personal decisions.

Not only are minority adolescents at higher risk of health and behavioral problems during adolescence, but they also face several barriers in accessing care that are inadequately addressed within the health system, including no insurance, lack of knowledge about the medical system, and limited transportation [6]. An estimated 9 million adolescents are uninsured; those in low-income families, single-parent households, and Latino are at the highest risk for being medically uninsured [7–9]. Compared with their insured counterparts, uninsured adolescents were five times as likely to lack a usual source of care, four times as likely to have unmet health care needs, and twice as likely to go without physician contact during the course of a year [10]. In addition, outside sources of health information and education for high-risk teens are inconsistent. Health education depends on the quality of classroom teaching, the access to computers and the Internet, and the health education levels of the surrounding community, all affecting health literacy [11]. Latino youth are particularly vulnerable to the lack of available and culturally appropriate health education [12]. For minority teens who may not be able to depend on other means of information, the health care system has a critical role in delivering accurate and available adolescent health information. Yet, gaps have been identified between the health information that teens want from the health care system and what is received [13]. Latino students have higher pregnancy and STD (gonorrhea, chlamydia, syphilis, and HIV/AIDS) rates than White non-Hispanic youth [14].

Physicians have been given guidance on adolescent screening and counseling by the American Medical Association (AMA) General Adolescent Preventive Services (GAPS) [15]. GAPS addresses the developmental, physical, and psychosocial health and serves as a comprehensive framework for preadolescent and adolescent services for patients between 11 and 21 years of age. These guidelines outline which topics should be addressed at each developmental stage and coincide closely with the top risk behaviors assessed by the Centers for Disease Control (CDC) from the Youth Risk Behavior Survey [16]. GAPS exists to increase health promotion and reduces teen morbidity and mortality by addressing teen risk factors.

The purpose of our study was to examine the gaps between teen risk behaviors, identified in the course of baseline interviews for an NIH funded research study, and subsequent disclosure at health care visits, in this case the initiation of care for latent tuberculosis. This juxtaposition allowed for the exploration of providers' assessment and documentation of adolescent risk behavior and the extent of adolescent disclosure to providers against the revealed risk behaviors in the health-study setting. The providers included physicians, nurse practitioners (NP), and physician assistants (PA).

## 2. Methods

This was a cross-sectional study based on baseline data from a larger study (National Heart, Lung, and Blood Institute, National Institutes of Health, Grant no. 5RO1 HL068595), conducted in 2003–2009. The study was approved by the Institutional Review Board of San Diego State University. The larger study was a TB medication adherence trial that examined the difference in adherence to isoniazid (INH) for the 9-month recommended regimen between two groups of San Diego County high school students receiving peer counseling.

### 2.1. Participants

The participants in this study were public high school students aged from 13 to 19, in southern San Diego County, who tested positive for LTBI during school screenings. Participants agreed to INH therapy subsequently initiated with a San Diego-based health care provider, agreed to participate in the INH compliance study, and signed a medical release form granting access to their medical charts.

Out of the 540 adolescents eligible for the study, 285 (52.8%) were recruited, and 263 (92.3%) were retained for analyses. Other than the refusal to participate, other reasons for not enrolling or continuing in the trial included disclosure of pregnancy, leaving home, refusal by their physicians to prescribe INH treatment, or LTBI treatment already initiated in Mexico.

### 2.2. Inclusion and Exclusion Criteria

The parent study included participants with a positive tuberculin skin test who agreed to treatment and consent. For this analysis, we included only those participants attending at least one provider visit for the treatment for latent tuberculosis.

### 2.3. Recruitment

For participants under 18 years of age, parental written consent and subject assent were obtained. Participants who were 18 years old signed their own consent form. All participants and parents/guardians of minor participants were asked to sign a medical records release form and received the participants' bill of rights.

### 2.4. Data Collection

Following informed consent and assent and prior to seeing a physician to initiate INH, trained research assistants of the same gender as the participant administered baseline interviews. The baseline interviews were conducted in a private room in participants' homes. Information was gathered on demographic characteristics, acculturation, health care barriers, risk behaviors, parenting practices, past healthcare use, medication-taking behavior, social support, self-esteem, TB knowledge and exposure, and use of adherence aids for medical regimens. Interviews were approximately 90 minutes in duration. Breaks were given when needed. Not all participants had to answer every question as skip patterns were used when the screening question was negative.

Interviews took place in a location that was out of hearing range of anyone other than the participant and the interviewer and confidentiality was verbally emphasized. Participants received 20 dollars cash for the completion of their baseline interview. Signed medical records release forms were collected; medical records were requested 12 months after enrollment and obtained by mail or fax. The charts were reviewed by two independent study researchers, and data were abstracted. Due to variability in the number of visits provided by the providers and the practice of nurse-only visits for chemoprophylaxis refills, only the first visit was used in this analysis. The time period was generally short between the interviews and the first visit with the provider, less than 3 months.

### 2.5. Measures

#### 2.5.1. TB Study Interview

Participants were asked several demographic (age, country of birth, and education) and social (employment and school activities) questions. Behavioral risks were assessed by 260 questions addressing several health and risk behaviors, including diet, exercise, mental health, birth control, sexual activity, tobacco use, alcohol and other substance use, injury prevention, violence, and dental health. For the purpose of this report, only the 85 questions related to tobacco, alcohol, substance use, sexuality, exercise, and violence were included. Basic demographic data were obtained from parents during an interview shortly after recruitment. While the survey as a whole was not validated, the questions were based on 20 years of study questions in assessing adolescent risk behavior. The full questionnaire is available upon request.

### 2.6. Chart Review

After the first visit to the provider, medical charts for each participant were examined for the following items: (1) documentation of provider screening for high-risk health behavior, (2) documentation of provider taking any action such as counseling or referrals to specialists, laboratory tests, or prescriptions written as a result of any identified risky behavior, and (3) characteristics of the clinic, type of insurance, and provider type (e.g., MD, NP, PA) and specialty, if applicable. Charts were reviewed by a CA-licensed physician investigator; 10% of charts received a second review by a licensed RN/MPH investigator for quality assurance. Interrater reliability was over 90%.

### 2.7. Analysis

The baseline provider interview was reviewed for behavioral risk factors which the participant reported during the prior year. Charts were reviewed for health and risk behavior assessments, responses, and actions taken, with frequency calculations.

## 3. Results

### 3.1. Participant and Provider Practitioner Demographics

The majority of participants (96%) were identified as Hispanic, 45% were foreign-born, and 46% were male. Participants were reasonably well distributed across all four grades of high school, with a mean age of 15.9 years (range: 13–19 years). A wide range of yearly household income was reported by the parents, with a mean of $20,000–$29,999 dollars per year (range: from 0 to $50,000+ a year).

Medical records were available for a total of 232 (88%) participants. Valid data were available on 221; the other 11 records were incomplete and did not include a record of the appropriate visit. During their first LTBI visit, participants were primarily seen (74%) by physicians (MD/DO), with the remainder by nurse practitioners (NP) and physician assistants (PA). These providers represented a variety of primary care settings and locations. The majority were seen in federally qualified community health centers (73%), private practices (17%), the County health clinic (6.3%), and large group practices (5%); missing data and other settings accounted for 2%.

### 3.2. Overall Disclosure and Identification of Risk Behaviors

The 221 teens had revealed to the project staff a total of 399 risk behaviors: tobacco: 41 (20 females); alcohol: 162 (84 females); street drugs: 32 (20 females); sexual exposures: 41 (23 females); lack of exercise: 39 (31 females); and interpersonal violence 51 (18 females). The use of birth control was reported in 34 of participants (18 females) (Table 1). The only significant gender differences found were the risks of lack of exercise (females at greater risk) and violence (males at greater risk). The risk behaviors were distributed as follows: 8 participants (3.6%) reported zero risks, 66 reported one risk (30%), 72 (33%) reported two risks, 36 (16%) reported three risks, 23 (10%) reported four risks, 13 (6%) reported five risks, and 3 (1.4%) reported six or more. The charting documented screening for these risk behaviors varied from 3% for violence to 52% for tobacco. The screening and disclosure by risk category is provided below.

### 3.3. Behavioral Risk Factors: Tobacco, Alcohol, and Drugs

There were 41 (19%) (20 females) teenagers who reported tobacco use in their baseline interview to the research staff. An assessment of tobacco use was documented in 114 (52%) (59 females) of medical encounters, more than for any other substance. This higher level of assessment reflects the inclusion of tobacco use and exposure in some previsit vitals assessment done by the medical assistant. Of the 41 teenagers who reported tobacco use to the research staff, providers documented asking 21. Only one teen (a female) reported tobacco use to their providers, and this teen received counseling; two additional teens had generic tobacco cessation counseling documented.

Almost three-fourths (162; 73%) participants (84 females) reported alcohol use in the baseline TB study interview. Providers documented asking about alcohol use to 48 (22%) (25 females) participants and identified only five participants (3 females) who reported alcohol use, four of whom received counseling from their provider; two additional teens received nonspecific antialcohol advice. Of the 162 teenagers reporting alcohol use to the research staff, providers documented asking 32.

Interviews of teens by the project staff revealed a total of 32 (15%) (20 females) cases of current or past drug use, with the most commonly used substances being marijuana (20), diet pills (10), and inhalants (4). Drugs used by only one to two participants included methamphetamines, cocaine, speed, LSD, and ecstasy. More providers documented screening for drug use than alcohol use, occurring in 89 (40%) (45 females) of the 221 assessed office encounters; however, only one teen (a female) admitted drug use. Of the 34 who reported drug use to research staff, providers documented asking 10. Four teens (2 females) received targeted and/or generic counseling by the provider.

### 3.4. Behavioral Risk Factors: Sexual Health

In interviews with project staff, teens were asked about their histories of sex, vaginal sex, anal sex, and oral sex. A total of 41 (19%) (23 females) teens reported a sexual history: 37 reported vaginal sex, 1 reported anal sex, and 17 reported oral sex. Interestingly, participants appeared to be more forthcoming to providers about their sexual activity than their substance use. Of the 38 participants (23 females) who were asked about sex by their providers, eight (7 females) reported sexual activity. Providers took some type of action in all eight cases and did generic counseling in an additional participant. Of the 41 teens reporting sexual behavior to the research staff, providers documented asking 12.

Project staff collected data regarding birth control use for the teens that reported any type of sexual history. A total of 34 (15%) (18 females) participants reported a history of birth control use, with control methods including condoms (34), pills (5), and the long-acting progesterone shot (1). No participants reported use of a patch, implantable device, diaphragm, sponge, or spermicide. Of the 31 (19 females) participants asked about birth control, seven participants (6 females) reported birth control use to their providers, and providers documented an intervention in all of these cases.

### 3.5. Behavioral Risk Factors: Exercise

Providers assessed physical activity in 21 of the 221 encounters. Although 38 (17%) (31 females) teens admitted a grossly sedentary lifestyle to research staff (<20 min exercise/week), providers identified two sedentary youth (1 female); three received targeted and/or generic counseling.

### 3.6. Behavioral Risk Factors: Violence

The interview assessed violence, fights, or carrying weapons in the last 12 months. A surprisingly high number of teens, 51 (23%) (18 females), screened positive for violence, including fights and weapons. One teen reported carrying a gun, 10 carrying a knife, and eight reported carrying a club. Of the six participants (4 females) screened for violence by their providers, none admitted to a positive history of violence. Two (1 female) received generic, nonspecific counseling.

## 4. Discussion

This study confirmed the gap between adolescent risk behaviors and the disclosure of these behaviors to health professionals. Of the total of 399 risk behaviors reported to the research project staff by participating teens, only 24 risk behaviors were disclosed to the providers. All but six teens reported at least one risk factor to project staff at baseline. The only significant differences by gender in behaviors reported to project staff were the baseline exercise assessment, with females significantly more likely not to exercise, and violence, with males at greater risk. There were no differences in provider behavior by gender of patient.

Although the primary purpose of the office visit in this study was for initiating INH therapy, the presence of a teen in any clinic setting should prompt inquiry and appropriate counseling for risk behaviors. A recent study documented that only 38% of adolescents had received preventive care visits in the last two years, and Hispanics and low-income adolescents were the least likely to receive such visits [17]. In addition, these preventive services are often not covered during a routine well-child exam [18]. Thus, one cannot rely on the well-child exam alone to identify youth with high-risk behavior or to provide anticipatory guidance to teens. Recent efforts to provide more patient-centered care are supported by this study, suggesting that nonphysician staff may be appropriate to elicit sensitive medical information from adolescents.

A unique component of this study is the prior confidential assessment of self-disclosed risk behaviors to ethnically similar research staff by the participants. This enabled the comparison of the risk behaviors reported to trained research assistants in confidential settings with those elicited and reported to their providers and documented in their medical records.

Several barriers were identified in this study that prevented providers and participants from discussing behavioral risk and risk avoidance strategies. First, providers either failed to inquire about behavior in the majority of cases in all risk areas or properly failed to document their inquiry. Providers missed almost all of the teen alcohol use, missing an opportunity for counseling in a very young drinking group. Second, when participants were asked about risk behaviors by providers, they did not disclose their risk behaviors to their providers to the same degree as they did with project staff. This difference was most evident with substance use, including tobacco; however, teens were more forthcoming to providers about sexual activity and birth control. This may represent the teens' perceived benefit of discussing sexual health in a medical setting, such as access to family planning resources. The paucity of questioning is concerning given a recent report that low-income and minority children receive more counseling during routine care than other groups [18]. An additional factor, one that we were unable to assess by the chart review, was the opportunity for teens to speak privately and confidentially with their providers. A recent study found that Latino children were least likely to get confidential care [19]. This information was not available from the chart review.

Several steps can be done to increase the likelihood of disclosure by teens, thus creating opportunities for prevention and counseling. This study demonstrated a paucity of screening (or documentation) and subsequent counseling. Provider education and awareness campaigns may be necessary to improve routine risk behavior screening in all encounters, as we have done for immunization. Time issues may also play a role, with many competing priorities in the primary care visit. In order for teens to volunteer information about their own behavior, questions need to be asked in a nonjudgmental, confidential, and teen-friendly way. Although this study did not look at methods for questioning and provider interactions with teens, the difference in reports to researcher versus providers in this study suggests a deficiency in provider-patient rapport.

The limitations of this study include the accuracy of self-report and chart review. Chart review included not only the progress notes but also lab notes, problem lists, immunization lists, and other sources of information in the chart. In the project interviews, every attempt was made to obtain accurate self-report in a both a neutral manner and confidential setting by trained, bilingual research assistants. The chart-review data were obtained on the first TB-related visit, and there is certainly a chance that on subsequent visits, that were not part of the chart audit, providers did appropriately query about health risk behaviors. In addition, this project was limited to the behavior and practice of providers located in southern San Diego County located clinics and private practices and may not represent other providers' practices locally, regionally, or nationally. This study was not a prevalence study, and the population has some unique features.

Nonetheless, this study highlights the gaps between teens high-risk behavior, their disclosure, and subsequent provision of counseling, which are likely generalizable.

## 5. Conclusions

Adolescents engage in high-risk behaviors, and providers have the opportunity to provide evidence-based and effective counseling if these behaviors are identified. All contacts with the medical system should be used to discuss common behavioral risks, as recommended by the AMA and GAPS guidelines. Greater effort to employ sensitive and trusted providers (e.g., LVNs) to obtain more complete reports of risk practices should be considered as a routine process prior to physician, NP, or PA appointments. This study revealed multiple barriers to the identification of risk factors by physicians of high-risk teens. Physicians should be aware that their adolescent patients are often engaging in high-risk behaviors and that adolescents limit their disclosure of this information.

## Figures and Tables

**Table 1:** Comparison of reported risk behavior to researchers versus health care providers and provider screening and action (*n* = 221 participants).

		Tobacco	Alcohol	Street drugs	Sexual behavior	Birth control	Lack of exercise	Violence
	Example: tobacco	*n* (%)	*n* (%)	*n* (%)	*n* (%)	*n* (%)	*n* (%)	*n* (%)
Risk factor identified during baseline interview	Participant told Fiesta they use tobacco	41 (19) 20 females	162 (73) 84 females	32 (15) 20 females	41 (19) 23 females	34 (15) 18 females	39 (17) 31* females	51 (23)** 18 females
Screened for risk by provider	Provider asked participant about tobacco use	114 (52) 59 females	48 (22) 25 females	89 (40) 45 females	38 (17) 23 females	31 (14) 19 females	21 (10) 10 females	6 (3) 4 females
Positive screen	Participant told provider they used tobacco	1 1 female	5 3 females	1 1 female	8 7 females	7 6 females	2 1 female	0
Counseled by provider (includes teens receiving counseling with negative hx)	Provider counseled participant about cessation	3 1 female	6 3 females	4 2 females	9 6 females	7 4 females	3 2 females	3 1 female

*Significant for females having more reporting of low exercise, 2-tailed *t*-test for equality of means, and *P* < .0001.

**Significant for males having greater risk of reporting violence, 2-tailed *t*-test for equality of means, and *P* < .0001.
